# Production of Novel Antibiotics Zeamines through Optimizing *Dickeya zeae* Fermentation Conditions

**DOI:** 10.1371/journal.pone.0116047

**Published:** 2014-12-26

**Authors:** Lisheng Liao, Yingying Cheng, Shiyin Liu, Jianuan Zhou, Shuwen An, Mingfa Lv, Yufan Chen, Yanfang Gu, Shaohua Chen, Lian-Hui Zhang

**Affiliations:** 1 Guangdong Province Key Laboratory of Microbial Signals and Disease Control, College of Natural Resources and Environment, South China Agricultural University, Guangzhou, Peoples' Republic of China; 2 Institute of Molecular and Cell Biology, Agency for Science, Technology and Research (A*STAR), Singapore, Republic of Singapore; 3 Department of Biological Sciences, National University of Singapore, Singapore, Republic of Singapore; University Paris South, France

## Abstract

*Dickeya zeae* strain EC1 was recently shown to produce a new type of phytotoxins designated as zeamine and zeamine II, which are potent wide-spectrum antibiotics against Gram-positive and Gram-negative bacterial pathogens, suggesting their promising potential as clinical medicines. In this study, the optimized medium composition and culture conditions for biosynthesis of novel antibiotics zeamines have been established by using response surface methodology, largely increasing the yield of zeamines from original about 7.35 µg·mL^−1^ in minimal medium to about 150 µg·mL^−1^ in LS5 medium. The study identified the major factors contributing to zeamines production, which include nitrate, sucrose, asparaginate, mineral elements Mg^2+^ and K^+^, and optimized amount of phosphate. In addition, the results showed that overexpression of *zms*K in *D. zeae* strain EC1 could further increase zeamines yield to about 180 µg·mL^−1^ in LS5 medium. The findings from this study could facilitate further characterization and utilization of these two novel antibiotics, and also provide useful clues for understanding the regulatory mechanisms that govern *D. zeae* virulence.

## Introduction

Discovery of antibiotics is one of the landmark medical advances in human history, allowing treatment of infectious illnesses once commonly fatal. Especially since 1950s, a range of new antibiotics have been discovered and prepared for clinical use, presenting an array of feasibilities and choices in treatment of various types of microbial infections [1], [2]. However, wide clinical application of antibiotics has also caused an undesirable consequence, i.e., emergence of superbugs which could resist a range of conventional antibiotics [2], [3]. It has now been widely accepted that the emergence of antibiotics resistance is an inevitable and irreversible trend, which presses an urgent need to discover and develop new types of antibiotics and new strategies of infection control.

We showed recently that *Dickeya zeae* strain EC1, a plant bacterial pathogen that causes rice foot rot and maize stem rot diseases, produces a new type of antibiotics designated as zeamine and zeamine II [4], [5]. Zeamine II is a long chain aminated polyketide and zeamine shares the same polyketide structure as zeamine II with an extra valine derivative moiety conjugated to the primary amino group of zeamine II. These antibiotics showed potent microbicidal activities against a wide range of Gram-positive and Gram-negative bacterial pathogens including multidrug-resistant bacteria such as *Staphylococcus aureus* and *Pseudomonas aeruginosa* [4], but the mechanism of inhibitory action remains unknown. Two genes in *D. zeae* associated with the biosynthesis of zeamines have been cloned and characterized,among them, *zms*A encodes a multidomain polyketide synthase [5], and *zms*K encodes a nonribosomal peptide synthase containing only a condensation domain [6]. High performance liquid chromatography (HPLC) and mass spectrometry analyses showed that mutation of *zms*A abolishes the production of both zeamine and zeamine II [5], whereas deletion of *zms*K blockes the biosynthesis of zeamine only [6]. The above findings indicate that *zms*A is involved in synthesis of the polyketide chain and *zms*K is responsible for catalysis of the amide bond formation by using zeamine II as a substrate to generate zeamine. HPLC analysis showed that zeamine is the major product of *D. zeae* strain DZ1, accounting for about 60% of the total antimicrobial activity, and zeamine II contributes to about 40% of the total antimicrobial activity [6]. Interestingly, other bacterial species could also produce zeamines. A gene cluster encoding the biosynthesis of zeamine antibiotics has recently been characterized in *Serratia plymuthica* [7].

However, the yield of zeamine is low at the level of about 10 mg per liter bacterial culture [4], and the yield of zeamine II is even less than zeamine [6]. Chemical synthesis of zeamines appears to be a good challenge as the compounds contain four amino groups with stereochemistry unknown. In this study, we investigated the environmental factors and culture conditions which might affect zeamines production. As quantification of zeamine and zeamine II separately is tedious and costly, which requires lengthy solvent extraction, conventional column chromatography and HPLC separation [4], here we determined the total yield of zeamines produced by the bacterium under various conditions using a bioassay based semi-quantitative method described previously [5]. Our results showed that zeamines biosynthesis is influenced by a range of factors, including nitrogen source, carbon source, mineral elements, and phosphate. By response surface methodology (RSM) analysis we optimized three key variables, i.e., NH_4_NO_3_, sucrose, and phosphate (K_2_HPO_4_, KH_2_PO_4_), and established a chemically defined medium LS5 for large scale production of zeamines. Under the optimal conditions, *D. zeae* strain EC1 produced more than 20-fold higher amount of zeamines than that produced in the previously reported minimal medium (MM). In addition, we found that overexpression of *zms*K could further increase the total yield of zeamines. Our findings present a chemically-defined medium for large scale production of zeamines and, may also provide useful clues for understanding the role and mechanisms of zeamines as phytotoxins in the pathogen-host interactions.

## Materials and Methods

### Bacterial strains and cultivation

*Dickeya zeae* EC1 and the deletion mutant Δ*zms*K were described in previous studies [6]. *Escherichia coli* was routinely maintained at 37°C in LB medium (per liter contains 10 g Bacto tryptone, 5 g yeast extract and 10 g NaCl). All other bacterial strains were grown at 28°C in LB medium or YEB medium (per liter contains 10 g Bacto tryptone, 5 g yeast extract, 5 g sucrose, 5 g NaCl, and 0.25 g MgSO_4_·7H_2_O, pH 7.0) or minimal medium (MM) [(per litre contains 10.5 g K_2_HPO_4_, 4.5 g KH_2_PO_4_, 2 g (NH_4_)_2_SO_4_, 2 g mannitol, 2 g glycerol, 0.2 g MgSO_4_·7H_2_O, 5 mg FeSO_4_, 10 mg CaCl_2_, and 2 mg MnCl_2_, pH 7.0] as indicated. The composition of the optimized medium named LS5 in this study includes 9.25 g K_2_HPO_4_, 3.3 g KH_2_PO_4_, 1.4 g NH_4_NO_3_, 12.7 g sucrose, 1 g KCl, 1 g Asparaginate and 0.25 g MgSO_4_, pH 7.0, per liter. Antibiotics were added at the concentrations when required, ampicillin, 100 µg·mL^−1^; kanamycin, 100 µg·mL^−1^; gentamycin, 50 µg·mL^−1^.

To prepare stock cultures, a single EC1 colony was inoculated in YEB broth and grown overnight with shaking at 200 rpm on an orbital shaker, and the cultures were adjusted to OD_600_ = 1.5 and glycerol was added to a final concentration of 20% *v*/*v*. These glycerol stocks were frozen in liquid nitrogen and stored at −80°C for further usage. For analysis of zeamines production, the stock cultures were added to medium in 1∶100 ratio. Similarly, the glycerol stocks of *E. coli* DH5α, which is highly sensitive to zeamines and used as indicator strain in zeamines analysis [4], [6], were also prepared and kept at −80°C for further usage.

### Quantification of zeamines

The total amount of zeamines including zeamine and zeamine II was quantified by a microbial plate bioassay as described previously with minor modifications [6]. Briefly, the quadrate bioassay plate (diameter was 12 cm) was prepared by adding about 25 ml of LB agar medium, which, after solidification, was overlaid with 20 mL of 1% agarose, at about 50°C, containing 200 µL of the stock culture of the indicator strain *E. coli* DH5α. And the wells of 4 mm in diameter were punched in the plates. The aliquots of *D. zeae* cultures were collected at 36 h after inoculation unless otherwise indicated. After centrifugation at 12,000 rpm, 500 µL of bacterial supernatants were collected in an eppendorf tube and the remaining bacteria in the supernatants were killed by placing the tubes in a boiling water bath for 10 min. To each well on the bioassay plate, 40 µL of boiled supernatants were added and the plates were incubated at 37°C for 24 h before measuring the diameters of inhibition zone. Inhibition zone widths in the bioassay were converted to zeamines concentration including zeamine and zeamine II using the formula: Zeamines (unit)  = 0.5484e^0.886x^, with a correlation coefficient (*R* ^2^) of 0.9957, X is the width in millimetres of the growth inhibition zone surrounding each well.

#### Determination of minimum inhibition concentration (MIC)

The minimum inhibition concentrations (MIC) of zeamines were determined as described previously [4]. Briefly, 96-well plates containing 2-fold serial dilutions of zeamine and zeramine II, which were purified as described [6], were prepared separately with LB liquid medium. The fresh overnight LB culture of *E. coli* strain DH5α was then inoculated to the above plates after dilution to yield a final density of 10^6^ colony forming units (CFU) per ml, respectively. The plates were then incubated with gentle shaking at 37°C. After 24 h, the plates were collected to measure OD_600_. The MIC was defined as the lowest concentration of the antibiotic allowing no visible growth. The MIC assay was repeated twice with triplicate each time.

### Experimental design

Development of a defined medium for zeamine production was commenced with the minimal medium described previously [8], with sequential modifications as described in the sections of Results. Each tested medium (10 ml in a 50 ml centrifuge tube) was inoculated with 1% *v*/*v* of EC1 stock culture grown in YEB medium, and incubated at 100 rev·min^−1^ on an orbital shaker, and grown for 36 h or otherwise indicated. Zeamines production was quantified by microbial bioassay described in the previous section.

Based on statistical approaches, response surface methodology (RSM) was explored to optimize the critical factors and their interactions which significantly affect the zeamines production of *D. zeae* strain EC1. Optimization of the parameters for zeamines production of *D. zeae* strain EC1 was done by a central composite rotatable design (CCRD), which requires five levels (−1.68, −1, 0, 1, 1.68). The coded and encoded variables used in RSM design are listed in Table 1. The results were an experimental design of 17 experimental points, including three central points. The model proposed for predicting the values of response variable was a quadratic one and expressed according to the following equation Eq. (1):
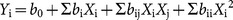
(1)where *Y*_i_ is the response variable, *b*_0_ is an intercept, *b*_i_ is the linear coefficients, *b*_ii_ is the quadratic coefficients and *b*_ij_ are the interactive coefficients. The analysis of the central composite experimental design was carried out using Design-Expert Software (Trial version 8.0.5b, Stat-Ease Inc., Minneapolis, MN).

**Table 1:** Uncoded and coded levels of independent variables of LS3 for zeamines production by *D. zeae* EC1.

Independent variables	Symbols	Range and level				
		−1.68	−1	0	1	1.68
NH_4_NO_3_ (mM·L^−1^)	*X*_1_	3.75	7.5	15	22.5	30
Sucrose (mM·L^−1^)	*X*_2_	7.5	15	30	45	60
Phosphate (mM·L^−1^)	*X*_3_	10.2	20.5	39	79	126.2

### Construction of *zmsK* over-expression strain

The over-expression vector of *zms*K is the same with the complementation vector of *zms*K, pBBR1-*zms*K, which was constructed following the methods described previously [7]. The over-expression construct pBBR1-*zms*K was transformed into *D. zeae* wild-type strain EC1 by triparental mating, in which donor *E. coli* DH5α (pBBR1-*zms*K) and recipient *D. zeae* EC1 were mixed with the helper strain HB101 (pRK2013) in a ratio of 2∶1∶1 on LB plate and incubated at 28°C overnight. The bacterial mixtures were diluted and spread on MM plates supplemented with 100 µg of ampicillin per milliliter to screen for transformants. The over-expression strain EC1(*zms*K) were confirmed by PCR analysis and DNA sequencing.

## Results

### Effect of nitrogen source on zeamines biosynthesis

**Figure 1:**
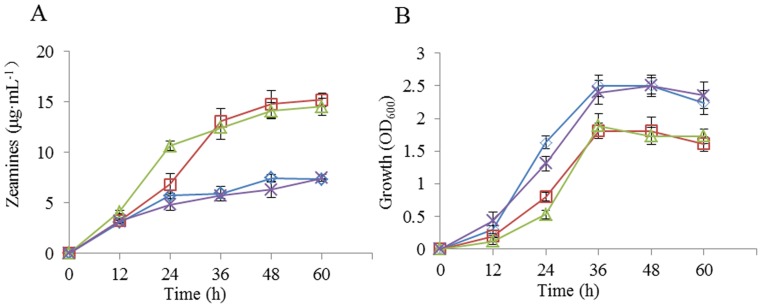
Effect of nitrogen source on zeamines biosynthesis (A) and growth (B) of *D. zeae* strain EC1. Symbol: (NH_4_)_2_SO_4_ (*white diamond*), NaNO_3_ (*white square*), NH_4_NO_3_ (*white triangle*), NH_4_Cl (*multiplication symbol*). Data are the means from four replicates per treatment.

In our previous studies, we noticed that minimal medium (MM) is superior than LB medium in supporting zeamines production of *D. zeae* strain EC1, and hence MM was used in zeamines preparation and analysis [4], [6]. Given that MM contains (NH_4_)_2_SO_4_ and LB contains tryptone as a nitrogen source, we firstly analyzed the effect of nitrogen source on zeamines production by using MM lacking nitrogen source as a basal medium. The semi-quantitative bioassay results showed that ammonium compounds (NH_4_)_2_SO_4_ and NH_4_Cl were the best nitrogen sources in supporting bacterial growth (Fig. 1B), whereas nitrate compounds NaNO_3_ and NH_4_NO_3_ performed much better than the former two ammonium compounds in supporting biosynthesis of zeamines (Fig. 1A) (Table 2). For the convenience of discussion, the MM media containing NaNO_3_ and NH_4_NO_3_ were designated as LS1 and LS2, respectively. As a control, zeamines production by *D. zeae* EC1 in LB medium was hardly detectable under the culture conditions used in this study (Table 2). In contrast, the bacterial pathogen produced about 3.56 µg zeamines per milliliter in YEB (Table 2). YEB is also a rich medium containing identical nitrogen source as LB but with extra sucrose as a carbon source, which may suggest a role of sucrose in supporting the biosynthesis of zeamines.

**Table 2:** Effect of nitrogen source on bacterial growth and zeamine production by *D. zeae* EC1.

Nitrogen source	OD_600_	Zeamines (µg·mL^−1^)	Significant difference
(NH_4_)_2_SO_4_	>2.5	7.35±0.70	B
NaNO_3_	1.73±0.10	15.20±1.23	A
NH_4_NO_3_	1.89±0.17	14.58±1.13	A
NH_4_Cl	>2.5	7.49±0.26	BC
Valine	1.78±0.11	0	D
Glycine	1.94±0.13	0	D
Tyrosine	1.98±0.21	0	D
(NH_4_)_2_SO_4_+Valine	1.83±0.18	7.0±1.41	BC
(NH_4_)_2_SO_4_+Tyrosine	1.67±0.21	5.65±1.36	C
LB	>2.5	0	D
YEB	>2.5	3.56±1.38	C

Note: Each inorganic nitrogen source was tested at 15 mM/L (Concentration of nitrogen molecules) and amino acid was tested at 1 g·L^−1^ in MM containing (per liter): 10.5 g K_2_HPO_4_, 4.5 g KH_2_PO_4_, 2 g mannitol, 2 g glycerol, 5 mg FeSO_4_, 10 mg of CaCl_2_, 2 mg MnCl_2_, 0.2 g MgSO_4_·7H_2_O, pH 7.0. Data are the means ± standard errors from three replicates per treatment at 36 h after inoculation. The multiple comparisons of means were obtained using Duncan's multiple-range test with an overall of 0.01. The means differing from each other were indicated with different capital letter (*P*<0.01).

*D. zeae* EC1 reached the exponential growth phase 12 h post inoculation and the bacterial growth was flattened at 36 h in either LS1 or LS2 (Fig. 1B). Concurrently, zeamines production in both media also entered into exponential phase at around 12 h after inoculation, but zeamines were continually produced even after bacterial growth was arrested (Fig. 1A).

### Effect of carbon source on zeamines biosynthesis

**Figure 2:**
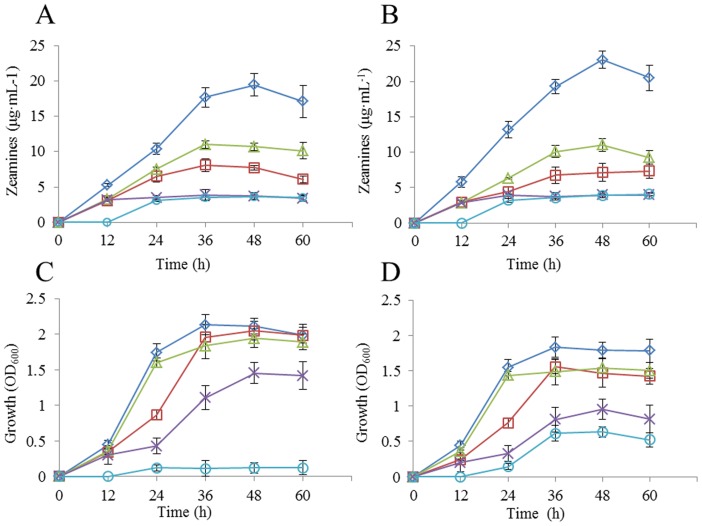
Effect of carbon source on zeamines production (A, B) and growth (C, D) of *D. zeae* strain EC1 in LS1 (A, C) and LS2 (B, D) media. Symbol: sucrose (*white diamond*), glucose (*white square*), mannitol (*white triangle*), glycerol (*multiplication symbol*), fructose (*white circle*). Data are the means from four replicates per treatment.

Similarly, by using the basal MM medium (containing 15 mM NaNO_3_ or 15 mM NH_4_NO_3_) lacking carbon source, we tested the effect of carbon sources on zeamines production. The results showed that sucrose was the best carbon source for zeamines production, followed by mannitol, glucose, glycerol and fructose, regardless whether NaNO_3_ or NH_4_NO_3_ was used as a nitrogen source (Fig. 2A, 2B; Table 3). It is interesting to note that while strain EC1 grew in a similar rate with either sucrose or glucose as sole carbon source (Fig. 2C), the organism produced over 2-fold higher amount of zeamines in LS1 containing sucrose than the same medium containing glucose (Fig. 2A, 2B) (Table 3). We also noted that supplementing sucrose in LS2 medium containing NH_4_NO_3_ was better than adding the same carbon molecule in LS1 containing NaNO_3_ in supporting zeamines production (Table 3). These findings suggest that sucrose and NH_4_NO_3_ could be an effective carbon and nitrogen combination in supporting zeamines production. The LS2 medium (lacking carbon source) containing sucrose was hence designated as LS3.

**Table 3:** Effect of carbon source on bacterial growth and zeamine production by *D. zeae* EC1.

Carbon source		OD_600_	Zeamines (µg·mL^−1^)	Significant difference
LS1	Sucrose	2.01±0.23	17.30±1.23	C
	Glucose	1.92±0.18	6.86±0.88	L
	Mannitol	1.73±0.09	9.51±1.81	I
	glycerol	0.86±0.31	3.31±0.22	P
	fructose	0.21±0.22	3.19±0.37	P
	Sucrose+Glucose	2.10±0.15	14.46±2.56	F
	Sucrose+Mannitol	1.98±0.27	15.34±3.11	E
	Sucrose+glycerol	1.82±0.22	8.34±1.21	J
	Sucrose+fructose	1.67±0.09	8.34±1.56	J
	Glucose+Mannitol	1.55±0.31	12.49±2.31	G
	Glucose+glycerol	1.69±0.12	4.66±1.01	NO
	Glucose+fructose	1.52±0.18	3.78±0.06	OP
	Mannitol+glycerol	1.93±0.16	7.35±0.21	K
	Mannitol+fructose	0.61±0.11	5.53±0.16	MN
	Glycerol+fructose	0.22±0.24	3.66±0.87	P
LS2	Sucrose	2.11±0.36	23.03±4.31	A
	Glucose	1.82±0.19	7.14±1.87	K
	Mannitol	1.66±0.21	11.03±1.05	H
	glycerol	0.86±0.17	3.93±1.32	OP
	fructose	0.61±0.08	3.92±1.43	OP
	Sucrose+Glucose	2.20±0.29	19.32±1.21	B
	Sucrose+Mannitol	1.91±0.31	19.62±1.88	B
	Sucrose+glycerol	1.72±0.12	16.45±0.97	CD
	Sucrose+fructose	1.67±0.11	10.34±0.14	HI
	Glucose+Mannitol	1.65±0.12	15.76±1.32	DE
	Glucose+glycerol	1.71±0.11	10.22±1.76	HI
	Glucose+fructose	0.92±0.16	9.39±2.44	I
	Mannitol+glycerol	1.93±0.39	5.64±1.32	LM
	Mannitol+fructose	0.67±0.24	5.77±1.65	LM
	Glycerol+fructose	0.86±0.15	3.66±0.77	P

Note: Each carbon source was tested at 5 g·L^−1^ in LS1 and LS2 medium. Data are the means ± standard errors from three replicates per treatment at 36h after inoculation. The multiple comparisons of means were obtained using Duncan's multiple-range test with an overall of 0.01. The means differing from each other were indicated with different capital letter (*P*<0.01).

### Effect of mineral elements on zeamines biosynthesis

*D. zeae* EC1 produced about 27.33 µg·mL^−1^ of zeamines when grown in LS3 medium that contains four mineral elements including Mg^2+^, Fe^2+^, Ca^2+^ and Mn^2+^ (Table 4). The bacterium failed to grow or produce zeamines when these mineral elements were eliminated from LS3 medium. Omission of MnCl_2_ alone from LS3 led to about 16% increase in zeamines production. Significantly, the yield of zeamines was increased by about 55% by removing both MnCl_2_ and FeSO_4_ from LS3 medium, and zeamines production was increased by 120% when only MgSO_4_ was left in the medium. Subsequent analysis showed that the best combination of mineral elements were Mg^2+^ and K^+^, and EC1 growing in the LS3 medium containing only these two elements produced about 63.69 µg·mL^−1^ zeamines, which is equivalent to about 133% of the yield in LS3 medium containing the four mineral elements (Table 4). The medium containing Mg^2+^ and K^+^ without other mineral elements was herewith designated as LS4.

**Table 4:** Effect of Mineral elements on bacterial growth and zeamines production by *D. zeae* EC1.

Mineral elements	OD_600_	Zeamine (µg·mL^−1^)	Significant difference
Mg^2+^+Fe^2+^+Ca^2+^+Mn^2+^	≥2.5	27.33±2.57	C
Mg^2+^+K^+^+Fe^2+^+Ca^2+^+Mn^2+^	≥2.5	24.17±2.86	C
Mg^2+^+K^+^+Fe^2+^+Ca^2+^	≥2.5	25.71±2.98	C
Mg^2+^+Fe^2+^+Ca^2+^	≥2.5	31.74±1.92	C
Mg^2+^+K^+^+Ca^2+^	≥2.5	43.04±3.02	B
Mg^2+^+Fe^2+^	1.499±0.04	12.85±1.69	D
Mg^2+^+K^+^	1.874±0.02	63.69±4.06	A
Mg^2+^+Ca^2+^	1.713±0.01	42.53±3.51	B
Mg^2+^	1.834±0.01	60.15±7.42	A
K^+^	0	0	E
Mg^2+^+K^+^(omitted)	0	0	E

Note: LS3 medium contains (per liter): 3.6 g NH_4_NO_3_, 10.5 g K_2_HPO_4_, 4.5 g KH_2_PO_4_, 15 g sucrose, 5 mg FeSO_4_, 10 mg of CaCl_2_, 2 mg MnCl_2_, 0.2 g MgSO_4_·7H_2_O, pH 7.0. Data are the means ± standard errors from three replicates per treatment. The multiple comparisons of means were obtained using Duncan's multiple-range test with an overall of 0.01. The means differing from each other were indicated with different capital letter (*P*<0.01).

### Effect of phosphate on zeamines biosynthesis

**Figure 3:**
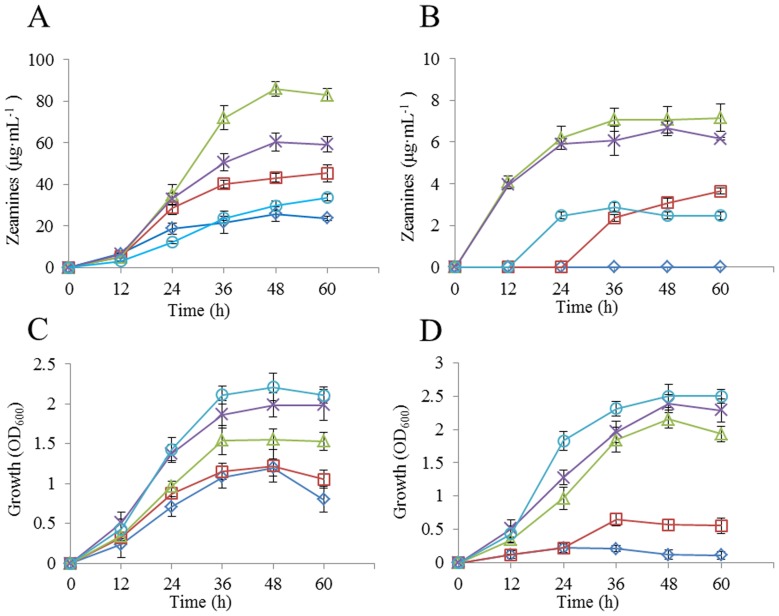
Effect of phosphate on zeamines production (A, B) and growth (C, D) of strain EC1 in LS3 (A, C) and MM (B, D) media. Following phosphate concentrations were tested: P1 (*white diamond*), 10.2 mM·L^−1^; P2 (*white square*), 20.5 mM·L^−1^; P3 (*white triangle*), 39 mM·L^−1^; P4 (*multiplication symbol*)), 79 mM·L^−1^; P5 (*white circle*), 126.2 mM·L^−1^. Data are the means from three replicates per treatment.

In LS4 and MM media, the total phosphate concentration is 79 mM (46 mM K_2_HPO_4_, 33 mM KH_2_PO_4_). To test the effect of phosphate on zeamines production, we proportionally change the ratio of K_2_HPO_4_ and KH_2_PO_4_ in these two medium to a final phosphate concentration of 10.2, 20.5, 39, 79 and 126.2 mM, respectively. The results showed that increasing the phosphate concentration in LS4 medium from 79 mM to 126.2 mM (Fig. 3A) increased the growth rate of *D. zeae* EC1 (Fig. 3C), but decreased the zeamines production by more than 50% (Fig. 3A). In contrast, zeamines production was increased by 42.5% when reducing the phosphate concentration to 39 mM (Fig. 3A).

Similarly, increasing phosphate concentration from 79 mM to 126.2 mM in MM medium did not substantially affect growth rate but almost completely suppressed the zeamines production (Fig. 3D). Reducing the phosphate concentration to 39 mM, zeamines biosynthesis was moderately increased by about 16%. EC1 failed to produce zeamines when phosphate concentration was reduced to 10.2 mM (Fig. 3B).

### Optimization of LS4 medium for zeamines production using RSM

Response surface methodology (RSM) has been widely used in medium optimization. In this study, RSM was applied to examine the effect of three medium components for enhancing zeamines production by EC1. A 20-run central composite rotatable design (CCRD) for three independent variables including NH_4_NO_3_ (*X*_1_), sucrose (*X*_2_) and phosphate (*X*_3_) were manipulated. The experimental values of zeamines production at different combinations of the independent variables were summarized in Table 1. The linear terms and the quadratic terms had the largest effect on EC1 zeamines yield, whereas the cross product term *X*_1_ *X*_3_ was not statistically significant (Table 5). After removal of the terms which were not statistically significant (on the basis of *p*-values which are more than 0.05), the model was rearranged in the following equation (Eq. 2):
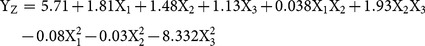
(2)where *Y*_z_ is the response, which is the yield of zeamines calculated with the second-order model, while *X*_1_, *X*_2_ and *X*_3_ are the coded values of independent variables.

**Table 5:** Analysis of variance (ANOVA) for the fitted quadratic polynomial model for zeamine production.

Source	SS		DF	MS	*F*-value	*P*-value
*X*_1_	89.94		1	89.94	8.54	0.0170
*X*_2_	241.75		1	241.75	22.94	0.0010
*X*_3_	585.61		1	585.61	55.58	<0.0001
*X*_1_ *X*_2_	143.51		1	143.51	13.62	0.0050
*X*_1_ *X*_3_	19.83		1	19.83	1.88	0.2033
*X*_2_ *X*_3_	6.03		1	6.03	0.57	0.4686
*X*_1_^2^	338.07		1	338.07	32.08	0.0003
*X*_2_^2^	770.09		1	770.09	73.09	<0.0001
*X*_3_^2^	1160.57		1	1160.57	110.15	<0.0001
Model	2026.76		9	225.20	21.37	<0.0001
Residual	94.83		9	10.54		
Lack of Fit	92.32		5	18.46	29.44	0.0030
Pure Error	2.51		4	0.63		
Total	2593.62		19			
*R*-squared = 0.9553		Adj *R*-Squared = 0.9106			Pred *R*-Squared = 0.4136	
CV % = 4.83		Adeq Precision = 14.042				

Note: SS – sum of squares. DF – degrees of freedom. MS – mean sum of squares. *P*-value <0.05 was considered significant.

The results of the response surface model described by Eq. (2) were given in Table 5 in the form of an analysis of variance (ANOVA). The ANOVA of the quadratic regression model demonstrated that the model is significant, as it can be observed from the Fisher's *F*-test (*F*model  = 21.4) with a low probability value (*P*<0.0001). The predicted versus observed values of total zeamines yield indicate a good agreement between the polynomial regression model and experimental data, with a coefficient of determination being 0.9532 (*R* ^2^), suggesting that only about 4.7% of the total variations can not be explained by the model. The value of adjusted determination coefficient (Adjusted *R* ^2^ = 0.9106) is also high, suggesting a high significance of the model. The low coefficient of variation (CV = 4.6%) also indicates that the model is accurate and reliable.

**Figure 4:**
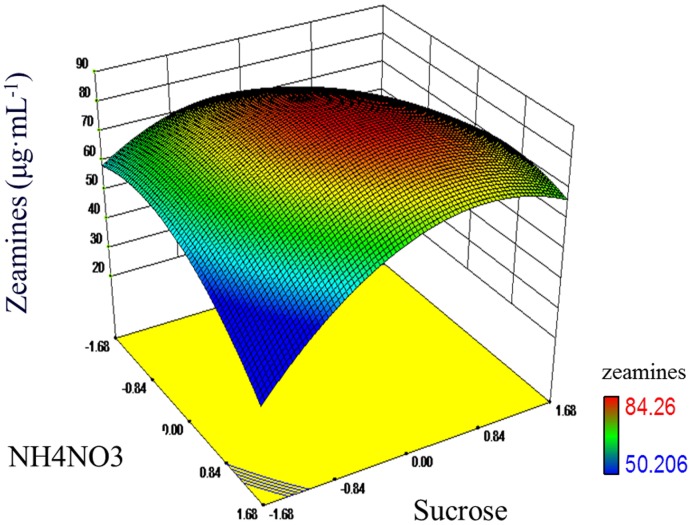
Response surface plot showing the effects of NH_4_NO_3_ and sucrose on zeamines production of strain EC1 with the value of phosphate being fixed at 64.67 mM·L^−1^. Data are the means from three replicates per treatment.

For better understanding of the results, the predicted model was presented in Fig. 4 as a 3-D response surface plot, which illustrates the effects of NH_4_NO_3_ and sucrose on zeamines production with phosphate as the constant. The model predicts a maximum zeamines production of 85.4 µg·mL^−1^ at the stationary point in the medium containing 17.12 mM NH_4_NO_3_, 37.09 mM sucrose, and 64.70 mM phosphate.

### Effect of amino acid and vitamin supplements in LS medium

Given that zeamine contain an amino acid derivative moiety, we tested the effect of amino acids on zeamines production. Twenty common amino acids were randomly divided into two groups to add separately into the LS4 medium. Zeamines production was increased by about 6.2% when LS4 supplemented with the first group of amino acids including asparagine, glutamic acid, valine, aspartic acid, serine, methionine, lysine, proline, threonine, and leucine (Table 6). However, adding the remaining ten amino acids caused about 12-fold decrease in zeamines production (Table 6). Subsequent experiments showed that addition of asparagine, glutamic acid, proline, aspartic acid and serine into LS4 could increase zeamines yield by 25.5%, whereas supplement of valine, methionine, lysine, threonine and leucine led to about 10-fold reduction in zeamines production (Table 6). Finally, we found that addition of asparaginate could increase zeamines production by about 50%. Similarly, addition of 0.1 g·L^−1^ yeast extract could also increase zeamines production by about 38% (Table 6). However, simultaneously addition of asparaginate and yeast extract in LS4 did not reveal any synergistic effect on zeamines production. LS4 medium containing 1 g asparaginate per liter was designated as LS5.

**Table 6:** Effect of amino acid and vitamin supplements on growth and zeamine production by *D.zeae* EC1 in LS4 medium.

Medium	OD_600_	Zeamine (µg·mL^−1^)	Significant difference
LS4	≥2.5	93.01±9.48	D
LS4+(Asparagine+Glutamicacid+Valine+Asparticacid+Serine+ Methionine+Lysine+Proline+ Threonine+Leucine)	≥2.5	98.87±7.67	DC
LS4+(Phenylalanine+Tryptophan+ Histidine+Tyrosine+Arginine + Isoleucine+Cystine+Glutarnine + Alanine+Glycine)	≥2.5	7.81±1.05	G
LS4+(Asparagine+Glutamicacid + Proline+Asparticacid+Serine)	≥2.5	117.16±14.04	BC
LS4+(Methionine+Lysine+Valine+ Threonine+Leucine)	≥2.5	8.93±0.69	G
LS4+Serine	≥2.5	53.85±4.11	E
LS4+Proline	≥2.5	100.13±7.64	DC
LS4+Leucine	≥2.5	29.45±5.76	F
LS4+Aspartic acid	≥2.5	122.97±13.23	AB
LS4+Valine	≥2.5	90.94±10.50	D
LS4+Asparaginate	≥2.5	139.71±27.24	A
LS4+Asparaginate+Valine	≥2.5	94.04±7.72	DC
LS4+Asparaginate +yeast extract	≥2.5	136.11±27.24	A
LS4+NH4NO3	≥2.5	98.87±7.67	DC
LS4+yeast extract	≥2.5	128.83±21.98	AB
LS4+Casein Hydrolysate	≥2.5	21.41±5.30	FG
LS4+tryptone	≥2.5	6.04±0.57	G

Note: Data are the means ± standard errors from three replicates per treatment. The multiple comparisons of means were obtained using Duncan's multiple-range test with an overall of 0.01. The means differing from each other were indicated with different capital letter (*P*<0.01).

### Effect of temperature and rotation speed

**Figure 5:**
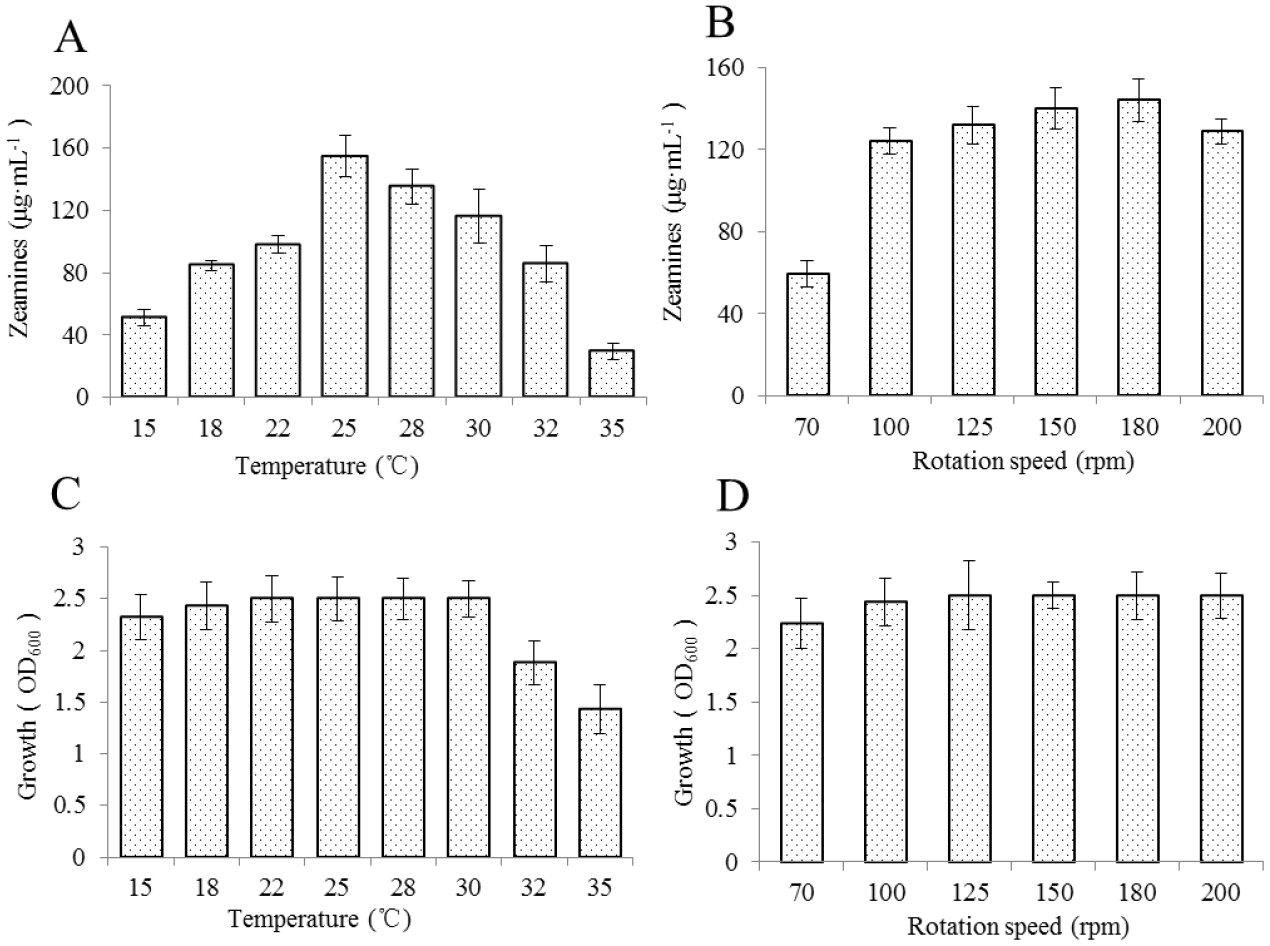
Effect of temperature (A, C) and rotation speed (B, D) on zeamines production (A, B) and growth (C, D) of strain EC1 inoculated in LS5. Data are the means from three replicates per treatment.

In our previous studies, we used 28°C for culturing bacteria in preparation of zeamines as we found that at this temperature EC1 produced higher amounts of zeamines than growing at 37°C. In this study, we tested the zeamines production amounts at range of temperatures from 15°C to 35°C. The results showed that 25°C was the best temperature for zeamines production with about 14% higher zeamines yield than that produced at 28°C (Fig. 5A). With increment of temperature, zeamines production of *D. zeae* EC1 was reduced progressively (Fig. 5A). Although zeamines production appeared to be sensitive to temperature changes, the bacterial growth was tolerant to temperatures ranging from 15°C to 30°C (Fig. 5C). However, temperature higher than 30°C could severely affect the bacterial growth (Fig. 5C).

Rotation speed of shaker in liquid culture may also influence bacterial growth and metabolism. So we tested EC1 growth and zeamines production amounts at different rotation speeds including 75, 100, 125, 150, 180, and 200 rpm, respectively. The experimental data showed that at 180 rpm the yield of zeamines was about 16% higher than that at the original shaking speed of 100 rpm and about 12% higher than that at the speed of 200 rpm (Fig. 5B). However, further reducing the shaking speed resulted in reduced production of zeamines (Fig. 5B). In contrast, variation of rotation speed did not seem to affect the bacterial growth rate (Fig. 5D).

### Over-expression of *zmsK* in *D. zeae* EC1 increases the total yield of zeamines

**Figure 6:**
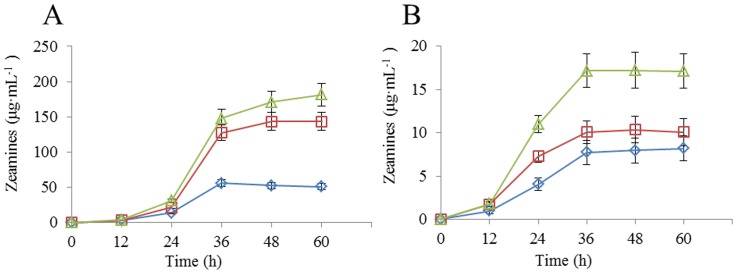
Effect of over-expression of *zmsK* on zeamines production. Three *D. zeae* strains including ΔzmsK (*white diamond*), EC1 (*white square*), and EC1(zmsK) (*white triangle*), were inoculated in LS5 (A) and MM (B) media, respectively. Data are the means from three replicates per treatment.

Above data suggest that the physical and chemical conditions could significantly affect the zeamines production of *D. zeae* EC1. To test whether we could further push the limit in zeamines yield, we generated a *zms*K overexpression construct by placing the coding sequence of *zms*K under the control of the *lac*Z promoter in the vector pBBR1-MCS4. We have shown previously that *zms*K encodes a nonribosomal peptide synthase that plays an essential role in zeamine biosynthesis [7]. The resulted over-expression construct was introduced into EC1 through triparental mating and the zeamines production was determined in LS5 and MM (minimal medium) media under the optimized conditions (25°C, 180 rpm), using *D. zeae* EC1 and the deletion mutant Δ*zms*K as controls. The results showed that EC1, Δ*zms*K and EC1(*zms*K) grew in a similar rate in both LS5 and MM media, but overexpression of *zms*K increased the total yield of zeamines by about 26.7% than the wild type EC1 (Fig. 6A). In MM medium, over-expression of *zms*K led to about 66.1% increment in the total yield of zeamines compared with EC1 (Fig. 6B). Considering that ZmsK converts zeamine II into zeamine [6], overexpression of *zms*K might substantially change the ratio of zeamine and zeamine II produced by *D. zeae* EC1, which awaits further investigations.

Interestingly, the total yield of zeamines produced by EC1 was almost 3 times of that produced by ΔzmsK in LS5 medium (Fig. 6A), whereas in MM (minimal medium) the zeamines yield of EC1 was only moderately higher than that produced by Δ*zms*K (Fig. 6B). Given that the deletion mutant Δ*zms*K produces only zeamine II but not zeamine [4], and the fact that zeamine and zeamine II showed an identical minimum inhibition concentration (MIC) against *E. coli* strain DH5α (0.5 µg.mL^−1^), the above findings seem to suggest that the optimized LS5 medium promote production of higher percentages of zeamine than the MM medium.

**Figure 7:**
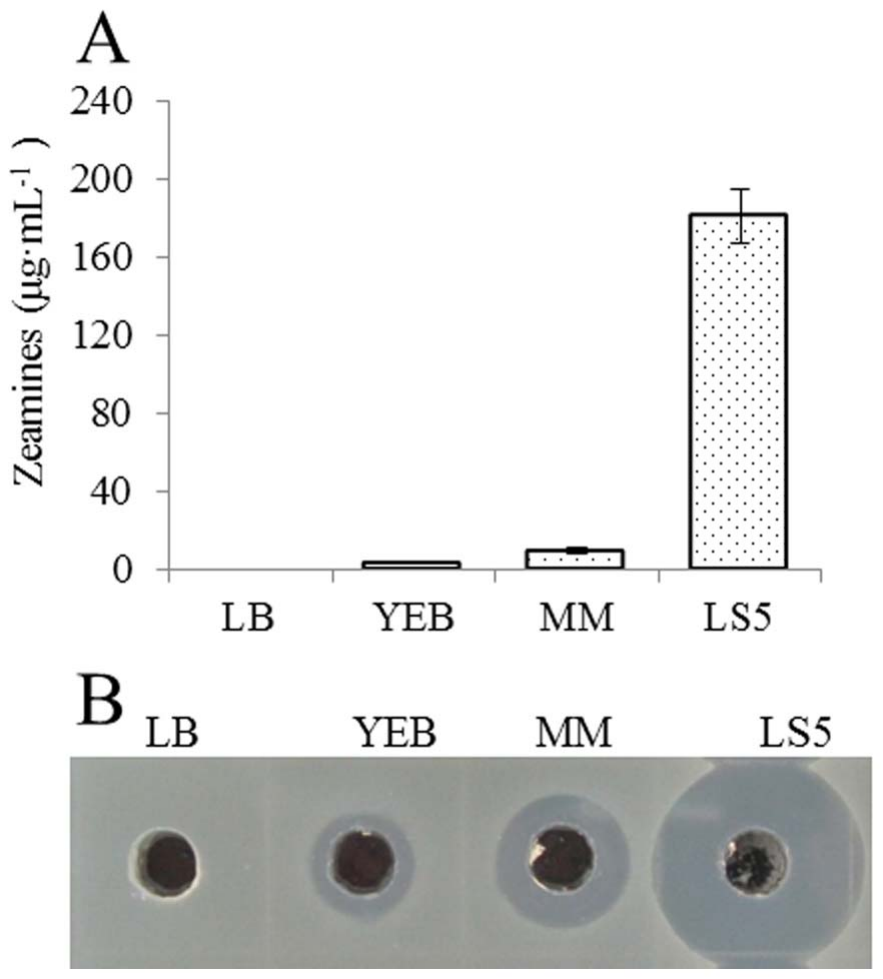
Zeamines production of strain EC1(*zmsK*) in different media. Quantitative analysis and plate assay were shown in (A) and (B), respectively. Data (A) are the means from four replicates per treatment.

Under the same culture conditions, we compared the total yield of zeamines by engineered strain EC1 (*zms*K) in various media. The results showed that LS5 was the best medium, followed by MM and YEB in supporting zeamines production (Fig. 7A). In particular, the total yield of zeamines in LS5 medium was about 23-fold higher than that in MM medium. Under the culture conditions used in this study, zeamines production in LB medium was hardly detectable (Fig. 7B).

## Discussion

Zeamines are novel polyamino-amide antibiotic metabolites of *Dickeya zeae* and the structures have been established by NMR and detailed MS analyses [4], and L-(l-^13^C) valine feeding experiment suggests that C-5′ amino isobutyl moiety is derived from valine. We hence speculated that valine may be important for *D. zeae* in production of zeamines. However, the total zeamines yield was either not affected or even decreased when valine was used as the sole nitrogen source or supplemented together with asparaginate (Table 2). Our results also showed that the effect of various amino acids on zeamines production was not associated with the bacterial growth rate as EC1 grew well when supplemented with amino acids (Table 2). The role of asparaginate in zeamines biosynthesis remains to be further investigated.

In bacterial cells, sucrose is generally digested by the enzyme invertase, resulting in a mixture of fructose and glucose [9], which are further metabolized through TCA cycle. Interestingly, however, we found that supplementation of both glucose and fructose in LS2 medium resulted in a substantial reduction in bacterial growth than the same medium containing glucose as sole carbon source (Table 3). It is also intriguing that sucrose was much better than glucose or fructose in supporting zeamines production regardless whether in LS1 or LS2 medium (Table 3). Evidence is accumulating that sucrose is not only a common carbon source but could also be a potent signal molecule in living organisms. For example, sucrose is a signal molecule in plant assimilate partitioning [10], and a range of genes that are associated with sucrose signal transduction and regulation have been characterized [11]–[15]. In addition, sucrose also plays a role in induction of *hrp* gene expression in plant associated bacterium *Pseudomonas syringae* [16], [17]. Comparatively, the signaling mechanism of sucrose in bacterial pathogens is poorly understood. Further investigation is needed to determine whether and how sucrose could act as a signaling molecule in modulation of zeamines production in *D. zeae*. Given that sucrose is abundant in crop plants and a key factor affecting zeamines biosynthesis, investigation of the role of zeamines and sucrose in host-pathogen interactions may provide useful information about the mechanisms of bacterial infection.

Response surface methodology (RSM) is an empirical statistics model that has eliminated the drawbacks associated with classical methods, and has proven to be successful and useful for optimization of secondary metabolite production [18]–[21]. RSM can also be used to explore the optimal conditions which could influence the metabolic responses of microorganisms [22]–[26]. We showed here that application of the statistical experimental design technique in this study could efficiently increase the yield of zeamines production and substantially reduce the experimental costs. In the present study, a squadratic polynomial model Eq.(2) was successfully developed, which could be used for optimization of zeamines production by *D. zeae* EC1. Under the optimized conditions, *D. zeae* strain EC1 produced more than 20-fold higher amount of zeamines than that produced in the previously reported MM medium. Using this defined medium will avoid problems associated with the low yield of zeamines at bacterial culture, and may facilitate the purification and application of zeamines. This defined medium could also assist the studies of the biosynthetic pathway and regulatory mechanisms of these interesting antibiotics and phytotoxins.

Environmental conditions are known to affect the pathogenic process of *D. zeae*. Plantation stage infection is a common feature of rice foot rot disease with the disease occurring often following rice seedling transplantation, because damaged rice roots could facilitate bacterial invasion [27]. The findings that zeamines biosynthesis is sensitive to nutrient conditions under *in vitro* conditions may also be relevant to the *in planta* pathogen-plant interactions, given that zeamines are the key factors associated with the pathogenesis of *D. zeae*. Studies in some monocotyledon plants showed that sugars, amino acids and organic acids are the main organic compounds [28]. However, the root fluid composition in rice is still unknown. Investigation of the nutrient composition in the root fluid of resistant and susceptible rice varieties under the conditions in favor of plantation stage infection or disease development may provide useful clues to understand the mechanisms of rice foot rot pathogenesis and disease resistance.

In conclusion, we investigated the environmental factors and culture conditions which might affect zeamines production in this study. Our results showed that zeamines biosynthesis was influenced by a range of factors, including nitrogen source, carbon source, mineral elements, and phosphate. By RSM analysis we optimized three key variables, i.e., NH_4_NO_3_, sucrose, and phosphate (K_2_HPO_4_, KH_2_PO_4_), and established a chemically defined medium LS5 for large scale production of zeamines. Under the optimized conditions, *D. zeae* strain EC1 produced more than 20-fold higher amount of zeamines than that produced in the previously reported minimal medium. In addition, we found that overexpression of *zmsK* could further increase the total yield of zeamines. The findings from this study could facilitate further characterization and utilization of these two novel antibiotics. Furthermore, our results may also provide useful clues for understanding the regulatory mechanisms that govern *D. zeae* virulence.

## References

[1] Powers JH (2004) Antimicrobial drug development-the past, the present, and the future. Clin Microbiol Infect 10:23–31. 15522037 10.1111/j.1465-0691.2004.1007.x

[2] Taubes G (2008) The bacteria fight back. Science 321:356–361. 18635788 10.1126/science.321.5887.356

[3] Livermore D (2004) Can better prescribing turn the tide of resistance? Nat Rev Microbiol 2:73–78. 15035011 10.1038/nrmicro798

[4] Wu J, Zhang HB, Xu JL, Cox RJ, Simpson TJ, et al (2010) 13C labeling reveals multiple amination reactions in the biosynthesis of a novel polyketide polyamine antibiotic zeamine from *Dickeya zeae* . Chem Commun (Camb) 46:333–345. 20024369 10.1039/b916307g

[5] Zhou J, Zhang H, Wu J, Liu Q, Xi P, et al (2011) A novel multidomain polyketide synthase is essential for zeamine production and the virulence of *Dickeya zeae* . Mol Plant Microbe Interact 24:1156–1164. 21899437 10.1094/MPMI-04-11-0087

[6] Cheng Y, An S, Liu X, Chang C, Zou Y, et al (2013) A non-ribosomal peptide synthase containing a stand-alone condensation domain is essential for phytotoxin zeamine biosynthesis. Mol Plant Microbe Interact 26:1294–1301. 23883359 10.1094/MPMI-04-13-0098-R

[7] Masschelein J, Mattheus W, Gao LJ, Moons P, Van Houdt R, et al (2013) A PKS/NRPS/FAS hybrid gene cluster from *Serratia plymuthica* RVH1 encoding the biosynthesis of three broad spectrum, zeamine-related antibiotics. PLoS One 8:e54143. 23349809 10.1371/journal.pone.0054143 PMC3547906

[8] Hussain MB, Zhang HB, Xu JL, Liu Q, Jiang Z, et al (2008) The acyl-homoserine lactone-type quorum-sensingsystem modulates cell motility and virulence of *Erwinia chrysanthemi* pv. *zeae* . J Bacteriol 190:1045–1053. 18083823 10.1128/JB.01472-07 PMC2223575

[9] Reid SJ, Abratt VR (2005) Sucrose utilisation in bacteria: genetic organisation and regulation. Appl Microbiol Biotechnol 67:312–321. 15660210 10.1007/s00253-004-1885-y

[10] Chiou TJ, Bush DR (1998) Sucrose is a signal molecule in assimilate partitioning. Proc Natl Acad Sci U S A 95:4784–4788. 9539816 10.1073/pnas.95.8.4784 PMC22568

[11] Kocal N, Sonnewald U, Sonnewald S (2008) Cell wall-bound invertase limits sucrose export and is involved in symptom development and inhibition of photosynthesis during compatible interaction between tomato and *Xanthomonas campestris* pv. *vesicatoria* . Plant Physiol 148:1523–1536. 18784281 10.1104/pp.108.127977 PMC2577280

[12] Lalonde S, Boles E, Hellmann H, Barker L, Patrick JW, et al (1999) The dual function of sugar carriers, transport and sugar sensing. Plant Cell 11:707–726. 10213788 10.1105/tpc.11.4.707 PMC144201

[13] Tognetti JA, Pontis HG, Martinez-Noel GM (2013) Sucrose signaling in plants: A world yet to be explored. Plant Signal Behav 8:e23316. 23333971 10.4161/psb.23316 PMC3676498

[14] Wind J, Smeekens S, Hanson J (2010) Sucrose: metabolite and signaling molecule. Phytochemistry 71:1610–1614. 20696445 10.1016/j.phytochem.2010.07.007

[15] Yang Z, Zhang L, Diao F, Huang M, Wu N (2004) Sucrose regulates elongation of carrot somatic embryo radicles as a signal molecule. Plant Mol Biol 54:441–459. 15284498 10.1023/B:PLAN.0000036375.40006.d3

[16] Rahme LG, Mindrinos MN, Panopoulos NJ (1992) Plant and environmental sensory signals control the expression of *hrp* genes in *Pseudomonas syringae* pv. *phaseolicola* . J Bacteriol 174:3499–3507. 1592805 10.1128/jb.174.11.3499-3507.1992 PMC206034

[17] Xiao Y, Lu Y, Heu S, Hutcheson SW (1992) Organization and environmental regulation of the *Pseudomonas syringae* pv. *syringae* 61 *hrp* cluster. J Bacteriol 174 (6) 1734–1741. 1548225 10.1128/jb.174.6.1734-1741.1992 PMC205773

[18] Bezerra MA, Santelli RE, Oliveira EP, Villar LS, Escaleira LA (2008) Response surface methodology (RSM) as a tool for optimization in analytical chemistry. Talanta 76:965–977. 18761143 10.1016/j.talanta.2008.05.019

[19] Gao H, Liu M, Liu J, Dai H, Zhou X, et al (2009) Medium optimization for the production of avermectin B1a by *Streptomyces avermitilis* 14-12A using response surface methodology. Bioresour Technol 100:4012–4016. 19356927 10.1016/j.biortech.2009.03.013

[20] Liu GQ, Wang XL (2007) Optimization of critical medium components using response surface methodology for biomass and extracellular polysaccharide production by *Agaricus blazei* . Appl Microbiol Biotechnol 74:78–83. 17086412 10.1007/s00253-006-0661-6

[21] Chen S, Hu Q, Hu M, Luo J, Weng Q, et al (2011) Isolation and characterization of a fungus able to degrade pyrethroids and 3-phenoxybenzaldehyde. Bioresour Technol 102:8110–8116. 21727000 10.1016/j.biortech.2011.06.055

[22] Chen S, Dong YH, Chang C, Deng Y, Zhang XF, et al (2013) Characterization of a novel cyfluthrin-degrading bacterial strain *Brevibacterium aureum* and its biochemical degradation pathway. Bioresour Technol 132:16–23. 23395753 10.1016/j.biortech.2013.01.002

[23] Chen S, Lai K, Li Y, Hu M, Zhang Y, et al (2011) Biodegradation of deltamethrin and its hydrolysis product 3-phenoxybenzaldehyde by a newly isolated *Streptomyces aureus* strain HP-S-01. Appl Microbiol Biotechnol 90:1471–1483. 21327411 10.1007/s00253-011-3136-3

[24] Chen S, Hu W, Xiao Y, Deng Y, Jia J, et al (2012) Degradation of 3-phenoxybenzoic acid by a *Bacillus* sp. PLoS One 7:e50456. 23226289 10.1371/journal.pone.0050456 PMC3511583

[25] Chen S, Luo J, Hu M, Geng P, Zhang Y (2012) Microbial detoxification of bifenthrin by a novel yeast and its potential for contaminated soils treatment. PLoS One 7:e30862. 22348025 10.1371/journal.pone.0030862 PMC3278408

[26] Liu SB, Qiao LP, He HL, Zhang Q, Chen XL, et al (2011) Optimization of fermentation conditions and rheological properties of exopolysaccharide produced by deep-sea bacterium *Zunongwangia profunda* SM-A87. PLoS One 6:e26825. 22096500 10.1371/journal.pone.0026825 PMC3214017

[27] Goto M (1979) Bacterial foot rot of rice caused by a strain of *Erwinia chrysanthemi* . Phytopathology 69:213–216.

[28] Dragisic MJ, Zivanovic BD, Maksimovic VM, Mojovic MD, Nikolic MT, et al (2014) Filter strip as a method of choice for apoplastic fluid extraction from maize roots. Plant Sci 223:49–58. 24767115 10.1016/j.plantsci.2014.03.009

